# *N*,*N*′-Bis(3-chloro­phen­yl)malonamide

**DOI:** 10.1107/S1600536811031333

**Published:** 2011-08-11

**Authors:** Vinola Z. Rodrigues, Sabine Foro, B. Thimme Gowda

**Affiliations:** a Department of Chemistry, Mangalore University, Mangalagangotri 574 199, Mangalore, India; b Institute of Materials Science, Darmstadt University of Technology, Petersenstrasse 23, D-64287 Darmstadt, Germany

## Abstract

The asymmetric unit of the title compound, C_15_H_12_Cl_2_N_2_O_2_, contains two independent mol­ecules. In both independent mol­ecules, the N—H bond in one of the amide fragments is *anti* to the *meta*-chloro group of the adjacent benzene ring and that in the other amide group is *syn* to the other *meta*-chloro group. Furthermore, in both mol­ecules, each amide group is almost coplanar with the adjacent phenyl ring, making dihedral angles of 10.5 (2) and 8.7 (2)° in one molecule and 9.0 (2) and 9.6 (2)° in the other. The planes of the amide groups are inclined at dihedral angles of 83.4 (1) and 87.4 (1)° in the two mol­ecules. In the crystal, mol­ecules are linked into a chain by inter­molecular N—H⋯O hydrogen bonds.

## Related literature

For our studies on the effects of substituents on the structures and other aspects of *N*-(ar­yl)-amides, see: Arjunan *et al.* (2004 ▶); Gowda *et al.* (2010 ▶); Saraswathi *et al.* (2011 ▶), on *N*-(ar­yl)-methane­sulfonamides, see: Gowda *et al.* (2007 ▶) and on *N*-chloro-aryl­sulfonamides, see: Gowda & Kumar (2003 ▶).
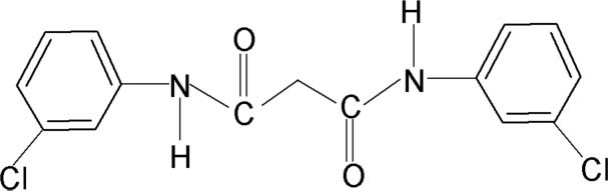


## Experimental

### 

#### Crystal data

C_15_H_12_Cl_2_N_2_O_2_
*M* *_r_* = 323.17
Monoclinic, 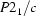

*a* = 10.9209 (8) Å
*b* = 16.416 (1) Å
*c* = 17.490 (1) Å
β = 105.260 (6)°
*V* = 3025.0 (3) Å^3^
*Z* = 8
Mo *K*α radiation
μ = 0.43 mm^−1^
*T* = 293 K
0.48 × 0.28 × 0.24 mm

#### Data collection

Oxford Diffraction Xcalibur diffractometer with a Sapphire CCD detector
Absorption correction: multi-scan (*CrysAlis RED*; Oxford Diffraction, 2009 ▶) *T*_min_ = 0.819, *T*_max_ = 0.903
12003 measured reflections
5164 independent reflections
2767 reflections with *I* > 2σ(*I*)
*R*_int_ = 0.027

#### Refinement

*R*[*F* ^2^ > 2σ(*F* ^2^)] = 0.047
*wR*(*F* ^2^) = 0.114
*S* = 0.96
5164 reflections
391 parameters
4 restraints
H atoms treated by a mixture of independent and constrained refinement
Δρ_max_ = 0.25 e Å^−3^
Δρ_min_ = −0.29 e Å^−3^

### 

Data collection: *CrysAlis CCD* (Oxford Diffraction, 2009 ▶); cell refinement: *CrysAlis RED* (Oxford Diffraction, 2009 ▶); data reduction: *CrysAlis RED*; program(s) used to solve structure: *SHELXS97* (Sheldrick, 2008 ▶); program(s) used to refine structure: *SHELXL97* (Sheldrick, 2008 ▶); molecular graphics: *PLATON* (Spek, 2009 ▶); software used to prepare material for publication: *SHELXL97*.

## Supplementary Material

Crystal structure: contains datablock(s) I, global. DOI: 10.1107/S1600536811031333/nc2242sup1.cif

Structure factors: contains datablock(s) I. DOI: 10.1107/S1600536811031333/nc2242Isup2.hkl

Supplementary material file. DOI: 10.1107/S1600536811031333/nc2242Isup3.cml

Additional supplementary materials:  crystallographic information; 3D view; checkCIF report

## Figures and Tables

**Table 1:** Hydrogen-bond geometry (Å, °)

*D*—H⋯*A*	*D*—H	H⋯*A*	*D*⋯*A*	*D*—H⋯*A*
N1—H1*N*⋯O3	0.84 (2)	2.11 (2)	2.950 (3)	176 (3)
N2—H2*N*⋯O4^i^	0.87 (2)	2.11 (2)	2.961 (3)	169 (3)
N3—H3*N*⋯O1^ii^	0.85 (2)	2.09 (2)	2.939 (3)	173 (2)
N4—H4*N*⋯O2	0.85 (2)	2.12 (2)	2.947 (3)	168 (3)

Symmetry codes: (i) 
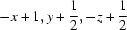
; (ii) 
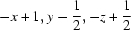
.

**Fig. 1.**
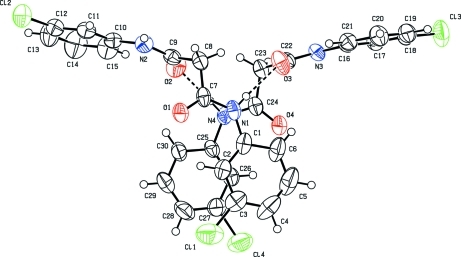
Molecular structure of the title compound, showing the atom labelling scheme with displacement ellipsoids drawn at the 50% probability level.

**Fig. 2.**
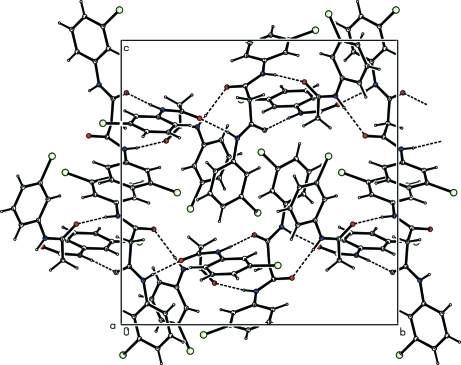
Crystal structure of (I) with hydrogen bonding shown as dashed lines.

## supplementary crystallographic information

### Comment

The amide and sulfonamide moieties are important constituents of many biologically significant compounds. As a part of studying the substituent effects on the structures and other aspects of *N*-(aryl)-amides (Arjunan *et al.*, 2004, Gowda *et al.*, 2010, Saraswathi *et al.*, 2011); *N*-(aryl)-methanesulfonamides (Gowda *et al.*, 2007) and *N*-chloro-arylsulfonamides (Gowda & Kumar, 2003), in the present work, the structure of *N*,*N*-bis(3-chlorophenyl)-malonamide (I) has been determined (Fig.1). The asymmetric unit of (I) contains two independent molecules. The conformations of all the N—H, C=O and C—H bonds in the central amide and aliphatic segments are *anti* to their adjacent bonds. Further, in both of the independent molecules, the N—H bonds in the amide fragments are *anti* to the *meta*-chloro groups in one of the adjacent benzene rings and *syn* to the *meta*-chloro group in the other, in contrast to the *syn* conformations of the N—H bonds with respect to the *meta*-methyl groups in the adjacent benzene rings of *N*,*N*-bis(3-methylphenyl)-malonamide (II)(Gowda *et al.*, 2010) and *anti* conformations of the N—H bonds with respect to the *meta*-chloro groups in *N*,*N*- bis(3-chlorophenyl)-succinamide (III) (Saraswathi *et al.*, 2011).

In the geometry of the molecule, each amide group is almost coplanar with the adjacent phenyl rings, as indicated by the dihedral angles of 10.5 (2)°, 8.7 (2)° (molecule 1) and 9.0 (2)°, 9.6 (2)° (molecule 2), compared to the value of 9.2 (2)° in (II). The planes of amide groups are inclined at angles of 83.4 (1)° (molecule 1) and 87.4 (1)° (molecule 2), in contrast to the value of 68.5 (1)° in (II). The phenyl rings of the two molecules make a dihedral angle of 21.5 (1)°.

In the crystal, the molecules are linked into chains by intermolecular N–H···O hydrogen bonding as shown in Fig. 2 (Table 1) .

### Experimental

Malonic acid (0.3 mol) in dichloromethane (30 ml) was treated with *m*-chloroaniline (0.6 mol) in dichloromethane (30 ml), dropwise with stirring. The resulting mixture was stirred for 3 hrs and kept aside for 12 hrs for the completion of reaction and evaporation of the solvent, dichloromethane. The product obtained was added to crushed ice to obtain the precipitate. The latter was thoroughly washed with water and then with saturated sodium bicarbonate solution and washed again with water. It was then given a wash with 2 N HCl. It was again washed with water, filtered, dried and recrystallized to the constant melting point from ethanol.

Prism like colorless single crystals of the title compound used in X-ray diffraction studies were obtained by a slow evaporation of its ehanolic solution at room temperature.

### Refinement

The H atoms of the NH groups were located in a difference map and later restrained to the distance N—H = 0.86 (2) Å. The other H atoms were positioned with idealized geometry using a riding model with the aromatic C—H = 0.93Å and the methylene C—H = 0.97 Å. All H atoms were refined with isotropic displacement parameters (set to 1.2 times of the *U*_eq_ of the parent atom).

### Crystal data

**Table:**

C_15_H_12_Cl_2_N_2_O_2_	*F*(000) = 1328
*M**_r_* = 323.17	*D*_x_ = 1.419 Mg m^−^^3^
Monoclinic, *P*2_1_/*c*	Mo *K*α radiation, λ = 0.71073 Å
Hall symbol: -P 2ybc	Cell parameters from 2892 reflections
*a* = 10.9209 (8) Å	θ = 2.7–27.8°
*b* = 16.416 (1) Å	µ = 0.43 mm^−^^1^
*c* = 17.490 (1) Å	*T* = 293 K
β = 105.260 (6)°	Prism, colourless
*V* = 3025.0 (3) Å^3^	0.48 × 0.28 × 0.24 mm
*Z* = 8	

### Data collection

**Table:**

Oxford Diffraction Xcalibur diffractometer with a Sapphire CCD detector	5164 independent reflections
Radiation source: fine-focus sealed tube	2767 reflections with *I* > 2σ(*I*)
graphite	*R*_int_ = 0.027
Rotation method data acquisition using ω and φ scans	θ_max_ = 25.2°, θ_min_ = 2.7°
Absorption correction: multi-scan (*CrysAlis RED*; Oxford Diffraction, 2009)	*h* = −13→12
*T*_min_ = 0.819, *T*_max_ = 0.903	*k* = −19→18
12003 measured reflections	*l* = −20→15

### Refinement

**Table:**

Refinement on *F*^2^	Primary atom site location: structure-invariant direct methods
Least-squares matrix: full	Secondary atom site location: difference Fourier map
*R*[*F*^2^ > 2σ(*F*^2^)] = 0.047	Hydrogen site location: inferred from neighbouring sites
*wR*(*F*^2^) = 0.114	H atoms treated by a mixture of independent and constrained refinement
*S* = 0.96	*w* = 1/[σ^2^(*F*_o_^2^) + (0.0536*P*)^2^] where *P* = (*F*_o_^2^ + 2*F*_c_^2^)/3
5164 reflections	(Δ/σ)_max_ < 0.001
391 parameters	Δρ_max_ = 0.25 e Å^−^^3^
4 restraints	Δρ_min_ = −0.29 e Å^−^^3^

### Special details

**Table:**

Geometry. All e.s.d.'s (except the e.s.d. in the dihedral angle between two l.s. planes) are estimated using the full covariance matrix. The cell e.s.d.'s are taken into account individually in the estimation of e.s.d.'s in distances, angles and torsion angles; correlations between e.s.d.'s in cell parameters are only used when they are defined by crystal symmetry. An approximate (isotropic) treatment of cell e.s.d.'s is used for estimating e.s.d.'s involving l.s. planes.
Refinement. Refinement of *F*^2^ against ALL reflections. The weighted *R*-factor *wR* and goodness of fit *S* are based on *F*^2^, conventional *R*-factors *R* are based on *F*, with *F* set to zero for negative *F*^2^. The threshold expression of *F*^2^ > σ(*F*^2^) is used only for calculating *R*-factors(gt) *etc*. and is not relevant to the choice of reflections for refinement. *R*-factors based on *F*^2^ are statistically about twice as large as those based on *F*, and *R*- factors based on ALL data will be even larger.

### Fractional atomic coordinates and isotropic or equivalent isotropic displacement parameters (Å^2^)

**Table:**

	*x*	*y*	*z*	*U*_iso_*/*U*_eq_	
Cl1	0.08136 (11)	0.24962 (7)	−0.09253 (6)	0.1097 (4)	
Cl2	1.03780 (9)	0.56633 (6)	0.20849 (5)	0.0876 (3)	
O1	0.42401 (19)	0.33751 (13)	0.14639 (11)	0.0623 (6)	
O2	0.6972 (2)	0.21642 (13)	0.22591 (12)	0.0640 (6)	
N1	0.3525 (2)	0.22476 (16)	0.19545 (14)	0.0479 (7)	
H1N	0.375 (3)	0.1947 (15)	0.2358 (12)	0.058*	
N2	0.7334 (2)	0.35055 (16)	0.25539 (13)	0.0464 (6)	
H2N	0.702 (2)	0.3931 (13)	0.2729 (15)	0.056*	
C1	0.2485 (3)	0.20088 (18)	0.13298 (19)	0.0495 (8)	
C2	0.2199 (3)	0.23552 (19)	0.05850 (18)	0.0541 (8)	
H2	0.2692	0.2777	0.0472	0.065*	
C3	0.1173 (3)	0.2066 (2)	0.0013 (2)	0.0684 (10)	
C4	0.0426 (3)	0.1446 (3)	0.0162 (3)	0.0912 (13)	
H4	−0.0271	0.1261	−0.0229	0.109*	
C5	0.0740 (4)	0.1104 (3)	0.0908 (3)	0.1005 (14)	
H5	0.0257	0.0675	0.1018	0.121*	
C6	0.1748 (3)	0.1384 (2)	0.1490 (2)	0.0751 (10)	
H6	0.1935	0.1154	0.1993	0.090*	
C7	0.4340 (3)	0.28635 (19)	0.19877 (17)	0.0454 (8)	
C8	0.5425 (3)	0.28865 (18)	0.27290 (15)	0.0506 (8)	
H8A	0.5345	0.2441	0.3077	0.061*	
H8B	0.5408	0.3394	0.3009	0.061*	
C9	0.6661 (3)	0.2813 (2)	0.24981 (15)	0.0459 (8)	
C10	0.8452 (3)	0.3654 (2)	0.23050 (15)	0.0439 (7)	
C11	0.8857 (3)	0.4459 (2)	0.23304 (15)	0.0504 (8)	
H11	0.8418	0.4864	0.2521	0.060*	
C12	0.9902 (3)	0.4656 (2)	0.20745 (16)	0.0574 (9)	
C13	1.0551 (3)	0.4076 (3)	0.1792 (2)	0.0786 (11)	
H13	1.1260	0.4212	0.1618	0.094*	
C14	1.0143 (4)	0.3290 (3)	0.1768 (2)	0.0896 (12)	
H14	1.0584	0.2893	0.1570	0.108*	
C15	0.9102 (3)	0.3058 (2)	0.20266 (19)	0.0660 (9)	
H15	0.8852	0.2515	0.2012	0.079*	
Cl3	0.23604 (9)	−0.20233 (6)	0.53548 (6)	0.0919 (4)	
Cl4	0.20833 (9)	0.00640 (8)	−0.10657 (5)	0.1024 (4)	
O3	0.4176 (2)	0.11744 (14)	0.33566 (12)	0.0667 (6)	
O4	0.4030 (2)	−0.01860 (13)	0.18439 (11)	0.0593 (6)	
N3	0.4492 (2)	−0.01203 (14)	0.38243 (12)	0.0413 (6)	
H3N	0.488 (2)	−0.0561 (13)	0.3785 (14)	0.050*	
N4	0.5293 (2)	0.08006 (14)	0.15599 (13)	0.0438 (6)	
H4N	0.586 (2)	0.1142 (14)	0.1768 (14)	0.053*	
C16	0.3616 (3)	−0.01776 (19)	0.42933 (13)	0.0394 (7)	
C17	0.3449 (3)	−0.09478 (19)	0.45764 (15)	0.0465 (8)	
H17	0.3916	−0.1387	0.4470	0.056*	
C18	0.2586 (3)	−0.1056 (2)	0.50165 (16)	0.0546 (8)	
C19	0.1891 (3)	−0.0411 (2)	0.51849 (17)	0.0622 (10)	
H19	0.1308	−0.0489	0.5481	0.075*	
C20	0.2076 (3)	0.0342 (2)	0.49082 (17)	0.0622 (9)	
H20	0.1612	0.0780	0.5022	0.075*	
C21	0.2933 (3)	0.04743 (19)	0.44636 (15)	0.0502 (8)	
H21	0.3049	0.0994	0.4282	0.060*	
C22	0.4710 (3)	0.0515 (2)	0.33970 (15)	0.0460 (8)	
C23	0.5658 (3)	0.03563 (18)	0.29160 (14)	0.0490 (8)	
H23A	0.6115	−0.0146	0.3087	0.059*	
H23B	0.6266	0.0799	0.2987	0.059*	
C24	0.4916 (3)	0.02930 (19)	0.20509 (16)	0.0448 (7)	
C25	0.4727 (3)	0.09431 (18)	0.07396 (15)	0.0429 (7)	
C26	0.3772 (3)	0.04626 (19)	0.02923 (17)	0.0506 (8)	
H26	0.3471	0.0016	0.0515	0.061*	
C27	0.3274 (3)	0.0660 (2)	−0.04923 (18)	0.0600 (9)	
C28	0.3689 (3)	0.1322 (3)	−0.08368 (19)	0.0762 (11)	
H28	0.3323	0.1451	−0.1365	0.091*	
C29	0.4653 (4)	0.1787 (2)	−0.03847 (19)	0.0762 (11)	
H29	0.4949	0.2235	−0.0609	0.091*	
C30	0.5188 (3)	0.15961 (19)	0.04018 (17)	0.0575 (9)	
H30	0.5856	0.1906	0.0702	0.069*	

### Atomic displacement parameters (Å^2^)

**Table:**

	*U*^11^	*U*^22^	*U*^33^	*U*^12^	*U*^13^	*U*^23^
Cl1	0.1013 (8)	0.1303 (10)	0.0822 (7)	0.0026 (7)	−0.0028 (6)	0.0018 (6)
Cl2	0.0953 (7)	0.0791 (8)	0.0886 (7)	−0.0246 (6)	0.0245 (5)	0.0113 (5)
O1	0.0801 (16)	0.0477 (15)	0.0581 (13)	−0.0170 (12)	0.0161 (11)	0.0143 (11)
O2	0.0779 (16)	0.0394 (15)	0.0806 (15)	−0.0013 (12)	0.0314 (12)	−0.0179 (12)
N1	0.0533 (17)	0.0379 (19)	0.0581 (17)	0.0049 (14)	0.0244 (14)	0.0116 (13)
N2	0.0567 (17)	0.0329 (19)	0.0520 (15)	0.0060 (14)	0.0183 (12)	−0.0084 (12)
C1	0.0450 (19)	0.033 (2)	0.076 (2)	0.0052 (16)	0.0267 (17)	0.0051 (17)
C2	0.049 (2)	0.045 (2)	0.072 (2)	−0.0011 (17)	0.0235 (16)	−0.0012 (18)
C3	0.057 (2)	0.059 (3)	0.084 (3)	0.006 (2)	0.0103 (19)	−0.004 (2)
C4	0.053 (2)	0.074 (3)	0.134 (4)	−0.014 (2)	0.002 (2)	−0.015 (3)
C5	0.070 (3)	0.074 (3)	0.155 (4)	−0.022 (2)	0.025 (3)	0.024 (3)
C6	0.059 (2)	0.056 (3)	0.111 (3)	−0.011 (2)	0.024 (2)	0.021 (2)
C7	0.055 (2)	0.033 (2)	0.0572 (19)	0.0008 (17)	0.0297 (16)	0.0018 (16)
C8	0.070 (2)	0.040 (2)	0.0474 (17)	−0.0036 (17)	0.0264 (15)	−0.0015 (15)
C9	0.059 (2)	0.040 (2)	0.0379 (16)	0.0043 (19)	0.0130 (14)	−0.0023 (15)
C10	0.0422 (18)	0.046 (2)	0.0416 (16)	0.0050 (17)	0.0085 (13)	−0.0045 (15)
C11	0.055 (2)	0.049 (2)	0.0460 (17)	0.0058 (18)	0.0122 (14)	−0.0066 (15)
C12	0.057 (2)	0.063 (3)	0.0501 (18)	−0.0036 (19)	0.0098 (15)	0.0052 (17)
C13	0.066 (3)	0.086 (3)	0.093 (3)	−0.003 (3)	0.037 (2)	−0.001 (2)
C14	0.076 (3)	0.082 (4)	0.129 (3)	0.019 (3)	0.058 (2)	−0.013 (3)
C15	0.067 (2)	0.046 (2)	0.091 (2)	0.0126 (19)	0.0323 (19)	−0.0072 (19)
Cl3	0.0972 (8)	0.0803 (8)	0.1077 (7)	−0.0137 (6)	0.0441 (6)	0.0323 (6)
Cl4	0.0661 (6)	0.1521 (11)	0.0806 (6)	−0.0219 (6)	0.0044 (5)	−0.0187 (6)
O3	0.0951 (17)	0.0433 (16)	0.0743 (14)	0.0199 (13)	0.0445 (12)	0.0203 (11)
O4	0.0681 (15)	0.0522 (16)	0.0596 (13)	−0.0158 (13)	0.0202 (11)	0.0084 (11)
N3	0.0560 (16)	0.0297 (17)	0.0407 (12)	0.0078 (13)	0.0174 (11)	0.0041 (12)
N4	0.0558 (17)	0.0353 (18)	0.0450 (15)	−0.0108 (12)	0.0218 (12)	−0.0038 (12)
C16	0.0478 (18)	0.041 (2)	0.0290 (14)	0.0003 (16)	0.0085 (12)	−0.0003 (14)
C17	0.0533 (19)	0.042 (2)	0.0432 (16)	0.0020 (16)	0.0102 (14)	0.0024 (15)
C18	0.056 (2)	0.059 (3)	0.0485 (17)	−0.0079 (18)	0.0140 (15)	0.0105 (16)
C19	0.057 (2)	0.082 (3)	0.0536 (19)	−0.006 (2)	0.0251 (15)	−0.002 (2)
C20	0.067 (2)	0.064 (3)	0.063 (2)	0.0073 (19)	0.0289 (17)	−0.0031 (19)
C21	0.061 (2)	0.043 (2)	0.0477 (17)	0.0008 (17)	0.0171 (15)	−0.0021 (15)
C22	0.057 (2)	0.040 (2)	0.0411 (16)	0.0048 (18)	0.0137 (14)	0.0046 (15)
C23	0.0547 (19)	0.047 (2)	0.0492 (17)	0.0036 (16)	0.0200 (14)	0.0108 (15)
C24	0.0523 (19)	0.038 (2)	0.0508 (18)	0.0014 (17)	0.0262 (15)	0.0021 (15)
C25	0.0553 (19)	0.040 (2)	0.0403 (17)	−0.0007 (16)	0.0255 (14)	−0.0032 (15)
C26	0.055 (2)	0.050 (2)	0.0515 (19)	0.0012 (17)	0.0233 (15)	0.0010 (16)
C27	0.0464 (19)	0.082 (3)	0.055 (2)	−0.0023 (18)	0.0182 (16)	−0.0085 (19)
C28	0.079 (3)	0.111 (4)	0.0435 (19)	0.002 (2)	0.0246 (19)	0.009 (2)
C29	0.097 (3)	0.088 (3)	0.052 (2)	−0.015 (2)	0.0347 (19)	0.011 (2)
C30	0.076 (2)	0.054 (2)	0.0502 (19)	−0.0157 (19)	0.0300 (16)	−0.0015 (17)

### Geometric parameters (Å, °)

**Table:**

Cl1—C3	1.734 (3)	Cl3—C18	1.734 (3)
Cl2—C12	1.731 (3)	Cl4—C27	1.723 (3)
O1—C7	1.226 (3)	O3—C22	1.223 (3)
O2—C9	1.224 (3)	O4—C24	1.225 (3)
N1—C7	1.338 (3)	N3—C22	1.340 (3)
N1—C1	1.409 (4)	N3—C16	1.418 (3)
N1—H1N	0.843 (16)	N3—H3N	0.853 (16)
N2—C9	1.343 (4)	N4—C24	1.337 (3)
N2—C10	1.421 (4)	N4—C25	1.424 (3)
N2—H2N	0.867 (17)	N4—H4N	0.845 (16)
C1—C6	1.377 (4)	C16—C21	1.381 (4)
C1—C2	1.380 (4)	C16—C17	1.387 (3)
C2—C3	1.376 (4)	C17—C18	1.376 (4)
C2—H2	0.9300	C17—H17	0.9300
C3—C4	1.372 (5)	C18—C19	1.379 (4)
C4—C5	1.378 (5)	C19—C20	1.363 (4)
C4—H4	0.9300	C19—H19	0.9300
C5—C6	1.367 (5)	C20—C21	1.383 (4)
C5—H5	0.9300	C20—H20	0.9300
C6—H6	0.9300	C21—H21	0.9300
C7—C8	1.509 (4)	C22—C23	1.518 (4)
C8—C9	1.513 (4)	C23—C24	1.520 (4)
C8—H8A	0.9700	C23—H23A	0.9700
C8—H8B	0.9700	C23—H23B	0.9700
C10—C15	1.372 (4)	C25—C26	1.376 (4)
C10—C11	1.391 (4)	C25—C30	1.382 (3)
C11—C12	1.370 (4)	C26—C27	1.375 (4)
C11—H11	0.9300	C26—H26	0.9300
C12—C13	1.356 (4)	C27—C28	1.376 (4)
C13—C14	1.362 (5)	C28—C29	1.370 (4)
C13—H13	0.9300	C28—H28	0.9300
C14—C15	1.383 (5)	C29—C30	1.382 (4)
C14—H14	0.9300	C29—H29	0.9300
C15—H15	0.9300	C30—H30	0.9300
			
C7—N1—C1	129.4 (3)	C22—N3—C16	128.3 (2)
C7—N1—H1N	111 (2)	C22—N3—H3N	116.7 (18)
C1—N1—H1N	119 (2)	C16—N3—H3N	114.8 (18)
C9—N2—C10	128.4 (3)	C24—N4—C25	128.6 (2)
C9—N2—H2N	116.6 (19)	C24—N4—H4N	116.9 (18)
C10—N2—H2N	114.8 (19)	C25—N4—H4N	113.6 (18)
C6—C1—C2	120.0 (3)	C21—C16—C17	120.0 (3)
C6—C1—N1	116.4 (3)	C21—C16—N3	123.9 (3)
C2—C1—N1	123.6 (3)	C17—C16—N3	116.0 (3)
C3—C2—C1	118.9 (3)	C18—C17—C16	119.4 (3)
C3—C2—H2	120.5	C18—C17—H17	120.3
C1—C2—H2	120.5	C16—C17—H17	120.3
C4—C3—C2	121.9 (3)	C17—C18—C19	121.1 (3)
C4—C3—Cl1	119.0 (3)	C17—C18—Cl3	119.3 (3)
C2—C3—Cl1	119.1 (3)	C19—C18—Cl3	119.6 (3)
C3—C4—C5	118.0 (4)	C20—C19—C18	118.7 (3)
C3—C4—H4	121.0	C20—C19—H19	120.6
C5—C4—H4	121.0	C18—C19—H19	120.6
C6—C5—C4	121.3 (4)	C19—C20—C21	121.8 (3)
C6—C5—H5	119.4	C19—C20—H20	119.1
C4—C5—H5	119.4	C21—C20—H20	119.1
C5—C6—C1	119.9 (4)	C20—C21—C16	118.9 (3)
C5—C6—H6	120.1	C20—C21—H21	120.5
C1—C6—H6	120.1	C16—C21—H21	120.5
O1—C7—N1	123.9 (3)	O3—C22—N3	124.7 (3)
O1—C7—C8	121.3 (3)	O3—C22—C23	120.3 (3)
N1—C7—C8	114.8 (3)	N3—C22—C23	115.0 (3)
C7—C8—C9	108.8 (2)	C22—C23—C24	107.5 (2)
C7—C8—H8A	109.9	C22—C23—H23A	110.2
C9—C8—H8A	109.9	C24—C23—H23A	110.2
C7—C8—H8B	109.9	C22—C23—H23B	110.2
C9—C8—H8B	109.9	C24—C23—H23B	110.2
H8A—C8—H8B	108.3	H23A—C23—H23B	108.5
O2—C9—N2	124.4 (3)	O4—C24—N4	124.2 (3)
O2—C9—C8	120.5 (3)	O4—C24—C23	120.7 (2)
N2—C9—C8	115.0 (3)	N4—C24—C23	115.1 (3)
C15—C10—C11	120.0 (3)	C26—C25—C30	120.6 (3)
C15—C10—N2	123.6 (3)	C26—C25—N4	122.8 (3)
C11—C10—N2	116.4 (3)	C30—C25—N4	116.6 (3)
C12—C11—C10	119.9 (3)	C25—C26—C27	118.3 (3)
C12—C11—H11	120.0	C25—C26—H26	120.9
C10—C11—H11	120.0	C27—C26—H26	120.9
C13—C12—C11	120.9 (3)	C28—C27—C26	122.3 (3)
C13—C12—Cl2	119.3 (3)	C28—C27—Cl4	118.6 (3)
C11—C12—Cl2	119.7 (3)	C26—C27—Cl4	119.1 (3)
C12—C13—C14	118.6 (3)	C29—C28—C27	118.6 (3)
C12—C13—H13	120.7	C29—C28—H28	120.7
C14—C13—H13	120.7	C27—C28—H28	120.7
C13—C14—C15	122.8 (4)	C28—C29—C30	120.5 (3)
C13—C14—H14	118.6	C28—C29—H29	119.8
C15—C14—H14	118.6	C30—C29—H29	119.8
C10—C15—C14	117.8 (3)	C25—C30—C29	119.7 (3)
C10—C15—H15	121.1	C25—C30—H30	120.1
C14—C15—H15	121.1	C29—C30—H30	120.1
			
C7—N1—C1—C6	−174.9 (3)	C22—N3—C16—C21	−8.5 (4)
C7—N1—C1—C2	6.2 (5)	C22—N3—C16—C17	170.5 (3)
C6—C1—C2—C3	0.0 (4)	C21—C16—C17—C18	1.0 (4)
N1—C1—C2—C3	178.9 (3)	N3—C16—C17—C18	−178.1 (2)
C1—C2—C3—C4	0.1 (5)	C16—C17—C18—C19	−0.4 (4)
C1—C2—C3—Cl1	−179.7 (2)	C16—C17—C18—Cl3	178.88 (19)
C2—C3—C4—C5	−0.7 (6)	C17—C18—C19—C20	−0.2 (4)
Cl1—C3—C4—C5	179.0 (3)	Cl3—C18—C19—C20	−179.5 (2)
C3—C4—C5—C6	1.4 (6)	C18—C19—C20—C21	0.2 (5)
C4—C5—C6—C1	−1.3 (6)	C19—C20—C21—C16	0.3 (4)
C2—C1—C6—C5	0.6 (5)	C17—C16—C21—C20	−0.9 (4)
N1—C1—C6—C5	−178.4 (3)	N3—C16—C21—C20	178.1 (3)
C1—N1—C7—O1	5.5 (5)	C16—N3—C22—O3	1.3 (5)
C1—N1—C7—C8	−174.1 (3)	C16—N3—C22—C23	−176.1 (2)
O1—C7—C8—C9	−60.1 (4)	O3—C22—C23—C24	−70.9 (3)
N1—C7—C8—C9	119.5 (3)	N3—C22—C23—C24	106.7 (3)
C10—N2—C9—O2	5.1 (5)	C25—N4—C24—O4	5.2 (5)
C10—N2—C9—C8	−172.8 (2)	C25—N4—C24—C23	−173.6 (3)
C7—C8—C9—O2	−71.9 (3)	C22—C23—C24—O4	−54.1 (4)
C7—C8—C9—N2	106.1 (3)	C22—C23—C24—N4	124.7 (3)
C9—N2—C10—C15	−6.8 (5)	C24—N4—C25—C26	−10.5 (4)
C9—N2—C10—C11	171.2 (3)	C24—N4—C25—C30	169.7 (3)
C15—C10—C11—C12	0.3 (4)	C30—C25—C26—C27	−1.3 (4)
N2—C10—C11—C12	−177.8 (2)	N4—C25—C26—C27	179.0 (3)
C10—C11—C12—C13	0.2 (4)	C25—C26—C27—C28	−0.8 (4)
C10—C11—C12—Cl2	178.4 (2)	C25—C26—C27—Cl4	179.7 (2)
C11—C12—C13—C14	−0.1 (5)	C26—C27—C28—C29	1.6 (5)
Cl2—C12—C13—C14	−178.3 (3)	Cl4—C27—C28—C29	−178.8 (3)
C12—C13—C14—C15	−0.6 (6)	C27—C28—C29—C30	−0.4 (5)
C11—C10—C15—C14	−0.9 (5)	C26—C25—C30—C29	2.5 (4)
N2—C10—C15—C14	177.0 (3)	N4—C25—C30—C29	−177.8 (3)
C13—C14—C15—C10	1.1 (6)	C28—C29—C30—C25	−1.6 (5)

### Hydrogen-bond geometry (Å, °)

**Table:**

*D*—H···*A*	*D*—H	H···*A*	*D*···*A*	*D*—H···*A*
N1—H1N···O3	0.84 (2)	2.11 (2)	2.950 (3)	176 (3)
N2—H2N···O4^i^	0.87 (2)	2.11 (2)	2.961 (3)	169 (3)
N3—H3N···O1^ii^	0.85 (2)	2.09 (2)	2.939 (3)	173 (2)
N4—H4N···O2	0.85 (2)	2.12 (2)	2.947 (3)	168 (3)

Symmetry codes: (i) −*x*+1, *y*+1/2, −*z*+1/2; (ii) −*x*+1, *y*−1/2, −*z*+1/2.
